# Phylloquinone and Menaquinone-4 Tissue Distribution at Different Life Stages in Male and Female Sprague–Dawley Rats Fed Different VK Levels Since Weaning or Subjected to a 40% Calorie Restriction since Adulthood

**DOI:** 10.3390/nu8030141

**Published:** 2016-03-04

**Authors:** Guylaine Ferland, Isabelle Doucet, Dominique Mainville

**Affiliations:** 1 Département de nutrition, Université de Montréal, Montréal, QC H3C 3J7, Canada; isadoucet@me.com (I.D.); dominique.mainville@hotmail.com (D.M.); 2 Hôpital de la Cité-de-la-Santé, Laval, QC H7M 3L9, Canada; 3 CIUSSS du Centre-Sud-de-l’Île-de-Montréal, Centre de réadaptation Lucie-Bruneau, Montréal, QC H2H 2N8, Canada

**Keywords:** phylloquinone, menaquinone-4, tissue distribution, sex, age, diet, caloric restriction

## Abstract

Whether through the vitamin K-dependent proteins or the individual K vitamers, vitamin K (VK) is associated with a number of age-related conditions (e.g., osteoporosis, atherosclerosis, insulin resistance, cognitive decline). In light of this, we investigated the influence of lifetime dietary VK exposure on the tissue distribution of phylloquinone (K_1_) and menaquinone-4 (MK-4) vitamers in 3-, 12- and 22-month-old male and female rats fed different K_1_ diets since weaning or subjected to a 40% calorie restricted diet (CR) since adulthood. Dietary K_1_ intakes around the minimal amount required for normal blood coagulation had no significant influence on body weights of both male and female rats at different life stages. Tissue contents of the K vitamers differed according to organs, were generally higher in females than in males, and increased with K_1_ intake. The MK-4/total VK ratios tended to be increased in old age possibly reflecting an increased physiological demand for MK-4 during aging. Our study also confirmed the greater susceptibility of male rats to low VK containing diet, notably at a younger age. Despite lifelong higher K_1_ intakes per unit body weight, tissue K_1_ and MK-4 contents at 20 months were generally lower in CR rats compared to their *ad libitum* (*AL)* counterparts. Whether the lower tissue MK-4 content is the result of lower synthesis from K_1_ or greater tissue utilization remains to be determined. However, the more youthful coagulation profile observed in old CR rats (*vs. AL* rats) tends to support the notion that CR is associated with greater utilization of the K vitamers to sustain physiological functions.

## 1. Introduction

As a unique cofactor to the gamma-glutamyl carboxylase (GGCX), an enzyme that converts specific glutamic acid residues to gamma-carboxyglutamic acid in precursor proteins, vitamin K (VK) *i.e.*, all K vitamers, participates in the biological activation of several proteins. The vitamin K-dependent proteins (VKDPs) are notably involved in hemostasis, the calcification process (*i.e.*, bone matrix and vasculature), brain function, and glucose metabolism [1,2,3,4]. Compounds with VK activity all have a common 2-methyl-1,4-naphtoquinone ring but differ in structure at the 3-position. Phylloquinone (K_1_), derived from plants, is the main dietary form, while the menaquinones, which are of bacterial origin, form a family of compounds with unsaturated isoprenyl side chains of various lengths [5]. One of the menaquinones, menaquinone-4 (MK-4), is not a common product of bacterial synthesis but is synthesized from phylloquinone with menadione as an intermediate [6,7,8]. In recent years, the human UbiA prenyltransferase containing 1 (UBIAD1) enzyme was shown to be responsible for the MK-4 biosynthesis [9,10].

In addition to its role as enzymatic cofactor in the carboxylation reaction, there is evidence to support other specific actions for VK notably, the K_1_ and MK-4 vitamers. Specifically, both vitamers have been involved in the synthesis of sphingolipids, a group of complex lipids present in brain cell membranes and which possess important cell signaling properties [11]. Recent studies also point to protective roles for K_1_ and MK-4 against oxidative stress and inflammatory processes. *In vitro*, both vitamers were shown to limit the production of interleukin-6 (IL-6) and other proinflammatory cytokines through the inhibtion of nuclear factor kappaB [12,13,14] whereas, *in vivo*, MK-4 limited inflammation in encephalomyelitis [15]. Both K_1_ and MK-4 have also been shown to prevent glutathione depletion-mediated oxidative injury and cell death in primary culture of oligodendrocyte precursors and immature fetal cortical neurons [16,17]. Menaquinone-4 specifically has also been shown to possess potent anticancer properties, inducing apoptosis in several types of tumor cells [18] and inhibiting growth of hepatocarcinoma cells [19]. At the molecular level, MK-4 is a ligand for the steroid xenobiotic receptor [20,21] and promotes protein kinase A (PKA) activation [22,23,24], a signaling pathway that has also been associated with enhanced testosterone production in rats [25].

Tissue concentrations of the K vitamers have been investigated in various rat strains and shown to vary according to organs [6,7,8], dietary intake [7,26,27,28], and sex [29,30]. The role of age on diet-induced changes in tissue VK distribution was also investigated in animals of various ages (*i.e.*, 3, 12, 24 months) fed different VK diets for short periods of time (about one month) [28,29]. Collectively, these studies have provided essential information on the factors that influence the tissue contents of the K vitamers in rats, and the mechanisms underlying the biotransformation of K_1_ to MK-4. However, to our knowledge, no studies have reported on lifetime VK dietary exposure on tissue distribution at distinct life stages.

Aging is a complex process that negatively impacts the development of the different systems and their ability to function. Age-related diseases include cancer, cardiovascular disease, diabetes, osteoporosis, and neurodegenerative diseases such as dementia and Alzheimer’s disease [31]. Caloric restriction (CR) is a well-established experimental model, which has been shown to increase longevity and delay the onset of age-related diseases in various experimental models (*i.e.*, invertebrate model organisms, rodents, primates). In rodents, this dietary regimen has been shown to extend lifespan by up to 50% [32,33].

Whether through the VKDPs or the specific actions of K_1_ and MK-4, VK is associated with numerous age-associated conditions including osteoporosis, diabetes, cardiovascular disease and cognitive decline [1,2]. In light of this, we endeavoured to investigate the influence of lifetime dietary VK exposure on the tissue distribution of the K_1_ and MK-4 vitamers in two models of aging. In the first study (Study 1), we report the tissue VK distribution in 3-, 12- and 22-month-old male and female rats fed different K_1_ diets since weaning. In the second study (Study 2), we report the VK tissue distribution in 20-month-old male and females rats, which were fed *ad libitum* (*AL*) throughout their lives or were subjected to a 40% calorie restricted diet (CR) since adulthood.

## 2. Methods and Materials

### 2.1. Animals and Diets

Protocols for both studies were approved by the Animal Care Committee of the Université de Montréal in compliance with the guidelines of the Canadian Council on Animal Care.

Study 1. A total of 120 rats were used for this study. 4-week-old male and female Sprague–Dawley rats were obtained from Charles Rivers Canada Inc. (St Constant, QC, Canada) and housed 3 per cage in suspended stainless steel wire-bottom cages (to prevent coprophagy) in a room kept at 22 °C with a 12 h light-dark cycle. Rats were kept in the same housing conditions and rat facility throughout the experimental period (22 months). For one week, animals had free access to a semi-synthetic powdered diet prepared according to the Teklad control diet (#TD 89248) which consisted of 22% casein (vitamin free), 48.5% sucrose, 15% corn starch, 5% corn oil, 4.6% fiber (cellulose), 3.9% mineral mix and 1% vitamin mix (Teklad Test Diets, Madison, WI, USA). After this acclimatization period, rats were randomly assigned to a diet containing either low (L; ~100 µg/kg diet), adequate (A; ~500 µg/kg diet) or high (H; ~1500 µg/kg diet) levels of K_1_. These diets compared to the Teklad control diet (#TD 89248) except for vitamin K, where menadione was replaced by K_1_ (Sigma Chemicals, St-Louis, MO, USA). The powder diets were prepared in our laboratory and K_1_ concentrations evaluated by HPLC analysis were: Mean ± SEM, *n* = 8; 104 ± 9 µg/kg diet, 433 ± 13 µg/kg diet, and 1668 ± 9 μg/kg diet, respectively. These dietary concentrations of K_1_ were chosen to provide rats with a range of intakes around the minimal amount required for normal coagulation activity namely 500 µg K_1_/kg diet [34]. Rats had free access to water and food. Food intake and body weights were recorded every 2 weeks, and the health of the rats was monitored regularly by a veterinarian throughout the experimental period. Distinct groups of male and female rats from each dietary group were killed at 3, 12 and 22 months of age (*n* = 5–7/sex/age/diet), after having been food deprived overnight prior to the day of the experiment.

Study 2. A total of 48 rats were used for this study. 4-week-old male and female Sprague–Dawley rats were obtained from Charles Rivers Canada, Inc. (St Constant, QC, Canada). Animals were individually housed in wire-bottom cages under constant temperature of 22 °C and a 12 h light/12 h dark cycle. After one week of acclimatization, rats were given free access to the Teklad control diet (#TD 89248) (described above) where menadione was replaced by K_1_ (Sigma Chemicals, St-Louis, MO, USA) at a concentration of ~500 µg/kg diet (Mean ± SEM, *n* = 8; 479 ± 13 µg K_1_/kg diet by analysis). At 8 months of age, 16 male and 16 female rats were randomized in two dietary groups: one group was given free access to food (*ad libitum* (*AL*)) while the second group was subjected to a 20% reduction in daily caloric intake for two weeks, and 40% (CR) thereafter based on the mean intake of *AL* rats. A gradual approach to CR was adopted to minimize stress on the animals. Diet fed the CR rats was adjusted for vitamins (including K_1_) and minerals, so that intakes of micronutrients were identical to those of *AL* animals: 22% casein (vitamin free), 48.5% sucrose, 15% corn starch, 5% corn oil, 4.6% fiber (cellulose), 5.85% mineral mix, 1.67% vitamin mix (Teklad Test Diets, Madison, WI, USA), and 835 µg K_1_/kg diet. Eight males and eight females aged three months and fed *AL* since weaning, served as the “young” control group. All animals had free access to water. Food intake and body weights were assessed weekly and every two weeks respectively, and health of the rats was monitored regularly by a veterinarian throughout the study. Distinct groups of male and female rats were killed at 3 and 20 (*AL* and CR) months of age (*n* = 6–8/group, after having been food deprived overnight prior to the day of the experiment.

### 2.2. Sacrifice Procedure (Applies to Both Studies)

On the day of sacrifice, rats were anesthesized with sodium pentobarbital (45 mg/Kg body weight, ip), blood was withdrawn from the abdominal aorta and collected in a vacutainer containing 9:1, *v*/*v* trisodium citrate (Becton Dickinson Co, Rutherford, NJ, USA), and plasma was immediately separated by a 10-min centrifugation (500× *g* at 4 °C). The liver, heart, kidneys, spleen, brain, ovaries or testis were harvested, washed in 0.9% saline solution, weighed and stored at −80 °C until analysis.

### 2.3. Analytical Procedure (Applies to Both Studies)

Phylloquinone and MK-4 were quantified by HPLC as previously described [35]. Briefly, tissue samples were pulverized in anhydrous Na_2_SO_4_ (10 times tissue weight) and extracted for 1 h in 10 mL of acetone containing an internal standard (2 ng/50 μL 2-Methyl-3-(3,7,11,15,19-pentamethyl-2-eicosenyl)-1,4-naphthalenedione—also referred to as (K_1_(25)) (GL Synthesis Inc., Worcester, MA, USA). Extracts were centrifuged (500× *g* at 4 °C for 10 min), dried under nitrogen at 45 °C and the solid residue was reextracted with 6 mL of hexane and 2 mL of water for 3 min. After centrifugation, the top hexane layer was dried under nitrogen and redissolved in 2 mL of hexane for solid phase extraction on 3 mL silica gel columns (JT Baker Inc., Phillipsburg, NJ, USA). The K_1_ and MK-4 fraction was eluted with 8 mL of hexane:diethyl ether (97:3, *v*/*v*), evaporated under nitrogen and the residue dissolved in 0.02 mL of dimethyl chloride and 0.180 mL methanol containing aqueous phase (10 mmol/L zinc chloride, 10 mmol/L acetic acid and 5 mmol/L sodium acetate; 5 mL aqueous phase was added to 1 L methanol). Quantitative analysis of the vitamers was performed by reverse-phase HPLC using a C-18 reverse phase column and fluorescence detection. The calibration standard consisted of a mixture of K_1_, MK-4 and K_1_(25) at 2 ng/50 μL. The percent recovery for the samples was calculated from the internal standard and found to be 85%–90%. Tissue concentrations are expressed relative to wet weight. Plasma K_1_ and MK-4 concentrations were quantified using the same HPLC technique as previously described [36,37].

Prothrombin times (PT) and activated partial thromboplastin times (APTT) were performed on an AC/100 Automated Clot Timer (Fisher Scientific Ltd, Montreal, QC, Canada) using thromboplastin and cephalin reagents (Sigma Chemical, St-Louis, MO, USA).

### 2.4. Statistical Analysis

All data are expressed as the mean ± SEM. Study 1. Body weight, food intake, K_1_ intake, PT, APTT, plasma and tissue K_1_ and MK-4 were analysed with respect to diet, sex and age, by three-way ANOVA (main effects) followed by pairwise multiple comparisons (Holm–Sidak method). Homogeneity of variance for ANOVA was assessed using the Shapiro–Wilk test and variables that were not normally distributed were log-transformed prior to the analysis. The effect of diet was further tested within each sex and age group by conducting distinct one-way ANOVA’s followed by Tukey’s *post hoc* tests. Tissue K_1_ and MK-4 concentration differences were tested in 12-month-old male and female rats fed the adequate diet (as reference groups), using one-way ANOVA’s followed by Tukey’s *post hoc* tests. Study 2. Body weight, food intake, K_1_ intake, PT, APTT, plasma and tissue K_1_ and MK-4 were assessed in males and females with respect to age and diet, with distinct one-way ANOVA’s followed by Tukey’s post hoc tests. Homogeneity of variance for ANOVA was assessed using the Shapiro-Wilk test and variables that were not normally distributed were log-transformed prior to the analysis. Student’ *t*-test was used to further assess the effect of CR at 20 months, and that of sex for given age and diet groups. Differences were considered significant at *p* < 0.05. Three-way analyses of variance were conducted using SigmaPlot version 12.0 (Systat Software, Inc., San Jose, CA, USA) whereas one-way analyses of variance and *t*-tests were conducted using GraphPad Prism6 version 6.01 (GraphPad Software, Inc., La Jolla, CA, USA).

## 3. Results

### 3.1. Study 1

#### 3.1.1. Body Weights, Food, and K_1_ Intakes

**Figure 1:**
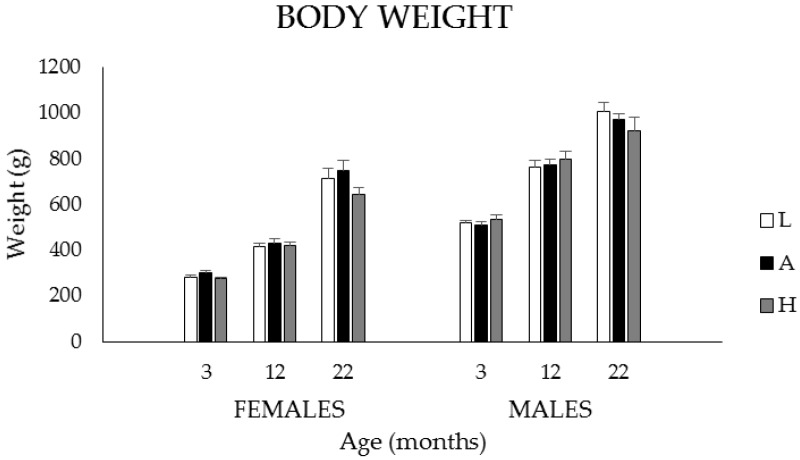
Mean body weights of 3-, 12-, and 22-month-old male (M) and female (F) Sprague–Dawley rats fed the L, A or H diets throughout their lives. Based on the 3-way ANOVA analysis, body weights increased with age in both M and F (*p* < 0.001). At all ages, body weights were higher in M than in F (*p* < 0.001) but were not affected by diet (*p* = 0.258), a finding confirmed by the individual one-way ANOVAs conducted at the group level. Values are expressed as mean ± SEM. (3 months F: *n* = 16/diet gp (group); M: *n* = 16/diet gp; 12 months F: *n* = 15–16/diet gp; M: *n* = 16/diet gp; 22 months F: *n* = 6–7/diet gp; M: *n* = 7–9/diet gp).

Mean body weights (BW) of male (M) and female (F) rats at 3, 12, and 22 months of age are presented in Figure 1 for the low (L), adequate (A) and high (H) dietary groups, respectively. Body weights increased with age in both males and females (*p* < 0.001). At all ages, body weights were higher in males than in females (*p* < 0.001) but were not affected by diet.

Daily food (g) and K_1_ (µg) intakes expressed per 100 g·BW are presented in Table 1 for 3-, 12-, and 22-month-old male and female rats fed the L, A or H diets. Food intakes decreased with age in both males and females (*p* < 0.001) and were higher in females than in males at 3- and 12- but not at 22-months of age (*p* < 0.001). Food intakes did not vary among the dietary groups in males at any age in contrast to females where, at 3 months, food intakes were significantly higher in the H than in the L and A groups (*p* < 0.05). Phylloquinone intakes decreased with age irrespective of diet in both males and females (*p* < 0.001), and were higher in females than in males at 3 and 12 but not at 22 months of age (*p* < 0.001). As expected, K_1_ intakes were significantly different between each dietary group, in both sexes (*p* < 0.001).

#### 3.1.2. Plasma and Tissue K_1_, MK-4, and Total VK (K_1_ + MK-4) Concentrations, and MK-4/Total VK Ratios

Plasma and tissue (the liver, heart, kidneys, spleen, brain, ovaries and testes) K_1_, MK-4 and total VK (K_1_ + MK-4) concentrations are presented in Table 2. In plasma, there were no main sex or age effects but in most groups, K_1_ concentrations increased with dietary K_1_ (F:H *vs.* A and L in all age groups; M:H *vs.* A and L at 12 months, H *vs.* L at 3 and 22 months (*p* < 0.05). No MK-4 could be detected in plasma hence group differences for total VK values are as those for K_1_.

Tissue K_1_ concentrations were highest in ovaries and liver and lowest in the brain and kidneys, other tissues presenting intermediate levels (*p* < 0.001). A main sex effect was seen for the liver, kidneys, and heart, females showing higher K_1_ concentrations than males (*p* < 0.001). The 3-way ANOVA also revealed a main diet effect for most organs (*p* < 0.001), differences being more frequently observed between the L and H, and the A and H groups (*p* < 0.05); dietary group differences (one-way ANOVA) are detailed in Table 2. A significant main effect was observed for age in the liver, heart, kidneys, and ovaries but differences varied according to organs and dietary groups (*p* < 0.001). Specifically, in the liver, K_1_ concentrations increased between 3 and 12 months and were decreased at 22 months (*p* < 0.05). In the heart and kidneys, K_1_ concentrations at 3 and 12 months were generally similar and were increased at 22 months, the extent of the change varying according to sex and dietary groups (*p* < 0.05). Finally, in ovaries, concentrations were observed to increase throughout life in all dietary groups (*p* < 0.05).

Tissue MK-4 concentrations were highest in ovaries and testes followed by the brain, the lowest amounts being seen in the heart and kidneys (*p* < 0.001). A main sex effect was observed for the liver, heart, kidneys, and the brain with females showing higher concentrations than males (*p* < 0.001). In most cases, the sex effect was observed at all ages and tended to be more pronounced in the H group (*p* < 0.05).

As for K_1_, MK-4 tissue concentrations varied as a function of diet, differences being mainly observed between the L and H, and the A and H groups (*p* < 0.05); dietary group differences (one-way ANOVA) are detailed in Table 2. The 3-way ANOVA also revealed a main age effect in all organs, differences varying according to organs, sex (when applicable), and dietary groups (*p* < 0.001). In liver and ovary, concentrations increased throughout life in all dietary groups (*p* < 0.05) whereas in the heart, kidneys, spleen, and the brain, the extent of age-associated increases varied according to sex and dietary groups. In testes, concentrations were comparable at 3 and 12 months (for a given dietary group), and were significantly decreased at 22 months, mainly due to the lower concentrations of rats fed the A and H diets (*p* < 0.05).

Tissue total VK (K_1_ + MK-4) concentrations were highest in ovaries followed by testes and liver, concentrations in other organs being statistically similar (*p* < 0.001). Except for the spleen, a main sex effect was observed in all organs, females showing higher total VK concentrations than males (*p* < 0.001). In the liver, the sex effect was observed at all ages and in most dietary groups while in the heart, kidneys and the brain, sex effect was more pronounced at 3 and 12 months in the H diet (*p* < 0.05). A main diet effect was also seen in most organs (*p* < 0.001), differences being mainly observed between the L and H, and the A and H groups (*p* < 0.05); dietary group differences (one-way ANOVA) are detailed in Table 2. The 3-way ANOVA also revealed a significant main effect for age in all organs, differences varying according to organs, sex (when applicable), and dietary groups (*p* < 0.001).

Specifically, in the liver, total VK concentrations increased between 3 and 12 months and decreased in the second year of life, the age effect being more pronounced in females fed the A and H diets (*p* < 0.05). In spleen, total VK concentrations were increased at 22 months compared to 3 and 12 months, the effect being particularly marked in males fed the H diet (*p* < 0.05). In brain, in both sexes, concentrations were comparable at 3 and 12 months (for a given dietary group) and were decreased at 22 months, largely due to the lower concentrations of rats fed the H diet (*p* < 0.05). In ovaries, total VK contents increased throughout life, the extent and time of change depending on the dietary groups (*p* < 0.05). Finally, in testes, total VK concentrations were comparable at 3 and 12 months (for a given dietary group), and were significantly decreased at 22 months, mainly due to the lower concentrations of rats fed the A and H diets (*p* < 0.05).

**Figure 2:**
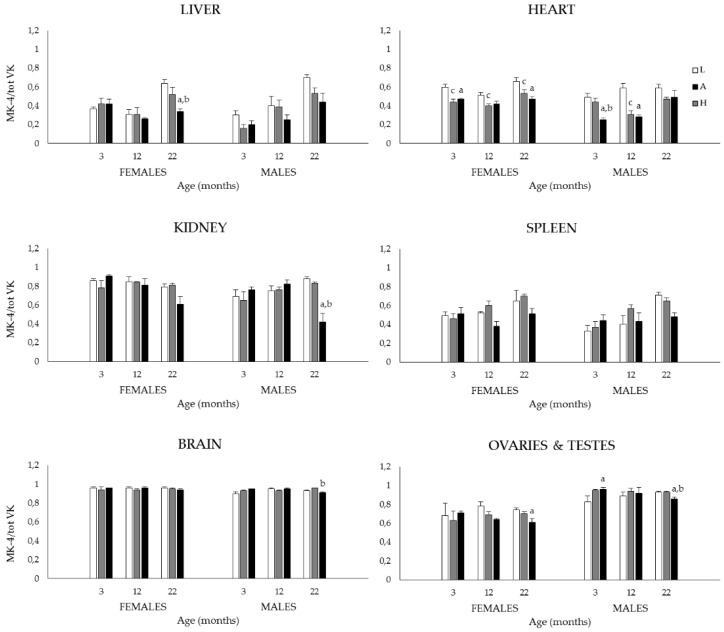
Tissue MK-4/total VK ratios of 3-, 12-, and 22-month-old M and F Sprague–Dawley rats fed the L, A or H diets throughout their lives. No ratios were computed for plasma as MK-4 could not be detected. Main sex, diet and age effects were assessed by conducting 3-way ANOVAs followed by pairwise multiple comparisons; results for these analyses are presented in the text (Section 3.1.2). The effect of diet on tissue MK-4/total VK ratios were further tested within each sex and age group by one-way ANOVA’s followed by Tukey’s *post hoc* tests; ^a^: *p* < 0.05 between H and L group same age and sex; ^b^: *p* < 0.05 between H and A group same age and sex; ^c^: *p* < 0.05 between A and L group same age and sex. Values are expressed as mean ± SEM; *n* = 5–7/sex/age/diet groups.

In light of the growing importance of MK-4 in various physiological functions, tissue MK-4 relative to total VK was computed for each organ in all animals as the ratio MK-4/total VK (Figure 2) (In plasma, MK-4 could not be detected hence no ratios were computed for this variable). In contrast to what is observed for K_1_, MK-4, and total VK, the MK-4/total VK ratios were much less affected by sex, diet, and age. Furthermore, MK-4 represents the main K vitamer in a number of organs. This is notably the case for the brain where the MK-4/total VK ratios are close to one irrespective of sex, diet and age. Menaquinone-4 is also the preferred K vitamer in testes and kidneys while ovaries, spleen, heart, and liver present more of a mixture of the two vitamers (*p* < 0.001). The 3-way ANOVA revealed a main sex effect in the heart, kidneys, and brain (*p* < 0.001), females showing higher ratios than males, (*p* < 0.05). The main diet effects were seen in all organs except for spleen (*p* < 0.001), ratios tending to be higher in the L diet groups (*p* < 0.05); dietary group differences (one-way ANOVA) are detailed in Figure 2. Finally, the 3-way ANOVA also revealed an age effect in liver, heart and spleen (*p* < 0.001). Specifically, in liver and spleen, ratios were increased at 22 months (*vs.* 3 and 12 months) notably in rats fed the L and A diets (*p* < 0.05). In the heart, ratios were increased at 22 months (*vs.* 3 and 12 months) especially in rats fed the A and H diets (*p* < 0.05).

#### 3.1.3. Prothrombin and Activated Partial Thromboplastin Times

**Figure 3:**
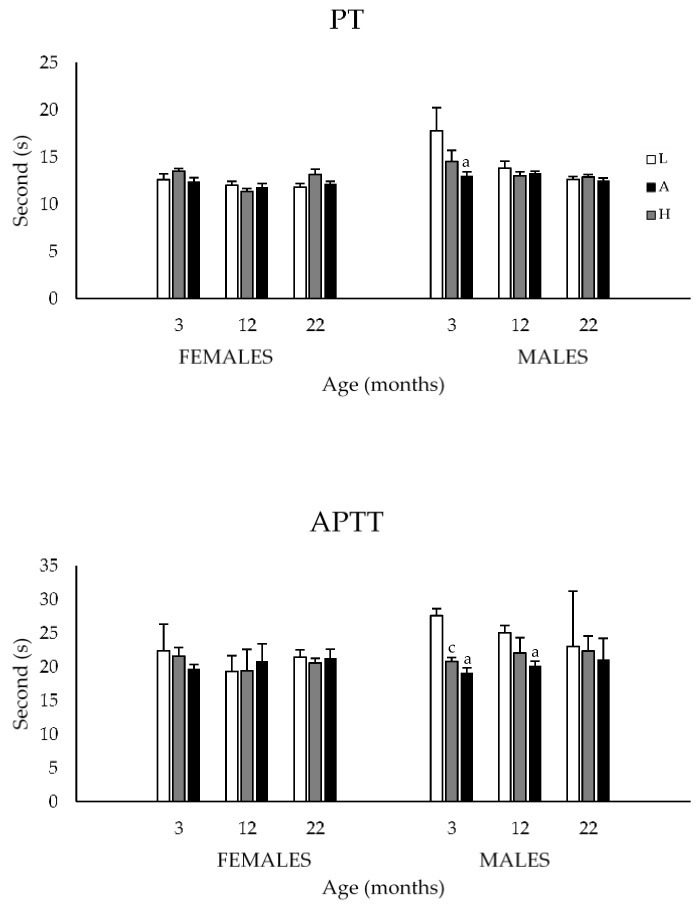
Prothrombin (PT) and activated partial thromboplastin (APTT) times of 3-, 12-, and 22-month-old M and F Sprague–Dawley rats fed the L, A or H diets throughout their lives. The 3-way ANOVA revealed no main sex and age effects for either variables. In contrast, a diet effect was observed in M rats fed the L diet. Specifically, this diet was associated with increased PT at 3 months and increased APTT at 3 and 12 months; ^a^: *p* < 0.05 between H and L group same age and sex; ^c^: *p* < 0.05 between A and L group same age and sex (one-way ANOVA). Values are expressed as mean ± SEM; *n* = 4–7/sex/age/diet groups.

Results for prothrombin (PT) and activated partial thromboplastin (APTT) times are presented in Figure 3. The 3-way ANOVA revealed no main sex and age effects for either variable. However, dietary K_1_ intakes significantly affected the coagulation times of male rats fed the L diet. Specifically, the low K_1_ containing diet was associated with increased PT in 3-month-old (*vs.* H diet), and increased APTT in 3- (*vs.* A and H diets) and 12-month-old (*vs.* H diet) male rats (*p* < 0.05).

### 3.2. Study 2

#### 3.2.1. Body Weights, Food, and K_1_Intakes

Body weights, daily food (g/day and g/100 g·BW/day), and K_1_ (µg/day and µg/100 g·BW) intakes of 3- and 20-month-old male and female Sprague–Dawley rats fed an *ad libitum* (*AL*) or calorie restricted (CR) diet are presented in Table 3. In all age and diet groups, body weights were significantly higher in male than female rats (*p* < 0.05). In animals of both sexes fed the *AL* diet, body weights were significantly increased at 20 months compared to 3 months and as expected, were signifiantly lower in the 20 CR than 20 *AL* group (*p* < 0.05).

Food intakes expressed as g/day were significantly higher in males than in females in all age and diet groups, and were higher in 20 *AL vs.* 3-month-old groups (*p* < 0.05). In both sexes and, as expected, food intakes of 20 CR rats were lower than those of the 20 *AL* group and also of the 3-month-old groups (*p* < 0.05). Phylloquinone (K_1_) intakes expressed as µg/day were significantly higher in males than in females (3 and 20 CR groups; (*p* < 0.05) but did not differ among age and diet groups.

Given the impact of the CR model on body weights, food (g) and K_1_ (µg) intakes are also reported per 100 g·BW. When expressed in this manner, food intakes were significantly higher in females than in males in the 3-month and 20 CR groups (*p* < 0.05), and were significantly lower in older animals (F: 20 *AL vs.* 3 months, 20 CR *vs.* 3 months; M: 20 *AL vs.* 3 months, *p* < 0.05). In addition, and in both sexeses, were lower in the 20 *AL vs.* 3-month-old group (*p* < 0.05).

Finally, when expressed as µg/100 g·BW/day, K_1_ intakes were significantly higher in females than in males in the 3-month and 20 CR groups (*p* < 0.05), and in both sexes, were lower in the 20 *AL vs.* 3-month-old group (*p* < 0.05). As mentioned previously, the diet fed the CR rats was adjusted to contain similar amounts of vitamins (including K_1_) and minerals as that fed the *AL* group. However, due to their lower body weights at 20 months, in both sexeses, K_1_ intakes (µg/100 g·BW/day) of CR rats were significantly higher than those fed the *AL* diet (*p* < 0.05).

#### 3.2.2. Plasma and Tissue K_1_, MK-4, and Total VK (K_1_ + MK-4) Concentrations, and MK-4/Total VK Ratios

Plasma and tissue (the liver, heart, kidneys, spleen, brain, ovaries and testes) K_1_, MK-4 and total VK (K_1_ + MK-4) concentrations of 3- and 20-month-old male and female Sprague–Dawley rats fed an *AL* or CR diet are presented in Table 4. In both sexes, plasma K_1_ concentrations were significantly increased at 20 (*AL* and CR) compared to 3 months, and were significantly higher in females than males (*p* < 0.05). A diet effect was also observed in both sexeses at 20 months, rats fed the CR diet showing decreased K_1_ concentrations compared to those of the respective *AL* groups (*p* < 0.05). Menaquinone-4 was undetectable in plasma, hence group differences for total VK values are as those for K_1_.

Tissue K_1_, MK-4, and total VK content profiles were similar to those reported in Study 1. Ovaries contained the highest amounts of total VK with high levels of both K vitamers (*p* < 0.001). Similarly, organs differed in their relative K_1_ and MK-4 contents (Table 4) with much higher MK-4/total VK ratios in organs such as the brain, testes, and kidneys (*i.e.*, 0.8–0.95) than in the liver and heart (*i.e.*, ~0.5).

Regarging K_1_, there was a general trend for concentrations to be higher in females than in males, this difference reaching statistical significance in kidneys (*p* < 0.05). In both sexes and for most organs, K_1_ concentrations were similar at 3 and 20 months except for ovaries where concentrations were significantly increased at 20 compared to 3 months (*p* < 0.05). Finally, and although differences reached statistical significance only in the liver (F) and kidneys (M) (*p* < 0.05), K_1_ concentrations at 20 months tended to be lower in the CR than in the *AL* groups (Table 4).

In contrast to K_1_, MK-4 concentrations were much more affected by sex with females showing higher concentrations than males in the liver (3 months), heart (20 CR), kidneys (all groups), spleen (20 *AL*), and the brain (3 months) (*p* < 0.05). In a number of organs, MK-4 concentrations also increased as a function of age, the effect being particularly marked in the liver (M: 20 *AL*, 20 CR), heart (F: 20 *AL*, 20 CR), kidneys (M: 20 *AL*, 20 CR; F: 20 *AL*), and spleen (M and F: 20 *AL*, 20 CR), (*p* < 0.05). Finally, as observed for K_1_, MK-4 concentrations tended to be lower in rats fed the CR regimen, differences reaching statistical significance in brain (F) and ovaries (*p* < 0.05) (Table 4).

As for the individual K vitamers, females generally presented higher tissue total VK concentrations than males, differences reaching statistical significance in kidney (all groups), spleen (20 *AL*), and brain (3 months) (*p* < 0.05). Similarly, total VK was generally increased at 20 months when compared to tissue concentrations at 3 months, the effect being more often observed in old rats fed the *AL* regimen liver (M: 20 *AL*, 20 CR), heart (M and F: 20 *AL*), kidney (M: 20 *AL*, 20 CR; F: 20 *AL*), spleen (F: 20 *AL*, 20 CR), and ovaries (20 *AL*). Finally, as oberved for individual K vitamers, the CR regimen was associated, at 20 months, with lower total VK concentrations, differences reaching statistical significance in ovaries (*p* < 0.05) (Table 4).

**Figure 4:**
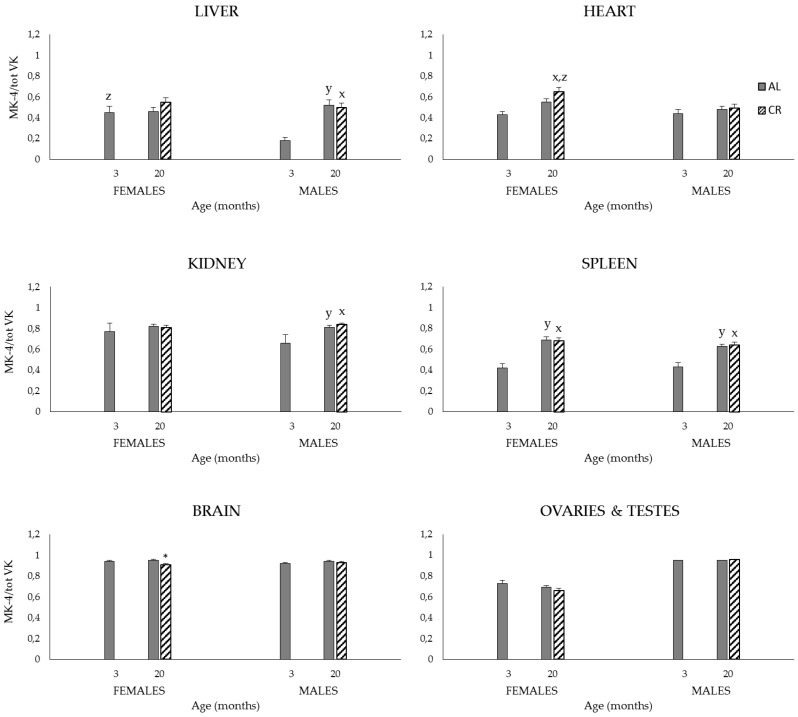
Tissue MK-4/total VK ratios of 3- and 20-month-old M and F Sprague–Dawley rats fed an *AL* or CR diet. Age and diet effects were determined in M and F by conducting distinct one-way ANOVA’s followed by Tukey’s *post hoc* tests. Student’ *t*-test was used to further assess the effect of CR at 20 months, and that of sex *i.e.*, given age and diet group. ^x^: *p* < 0.05 between 20 CR and 3 months, same sex; ^y^: *p* < 0.05 between 20 *AL* and 3 months, same sex; ^z^: *p* < 0.05 between M and F, same age and diet; *: *p* < 0.05 between 20 *AL* and 20 CR, same sex. Values are expressed as mean ± SEM; *n* = 6–8/sex/age/diet groups.

Finally, MK-4/total VK ratios were little affected by sex although they were significantly higher in females than males in liver (3 months) and heart (20 CR) (*p* < 0.05) (Figure 4). As observed in study 1, ratios were increased at 20 months in the liver (M: 20 *AL*, 20 CR), heart (F: 20 CR), kidneys (M: 20 *AL*, 20 CR), and spleen (M and F: 20 *AL*, 20 CR) (*p* < 0.05). Finally, MK-4/total VK ratios at 20 months were largely unaffected by the CR regimen with only the ratio for brain (F) being statistically decreased in CR compared to that in the *AL* group (*p* < 0.05).

#### 3.2.3. Prothrombin and Activated Partial Thromboplastin Times

Results for prothrombin (PT) and activated partial thromboplastin (APTT) times are presented in Table 5. At 20 months of age, a diet effect was observed in male rats, the mean PT value of CR rats being statistically increased compared to that of *AL* animals (*p* < 0.05), and comparable to that of rats aged 3 months. A sex effect was also observed for PT in 20-month-old rats fed the CR diet, males showing a statistically higher mean value than females (*p* < 0.05).

## 4. Discussion

All K vitamers classically serve as cofactors in a carboxylation reaction that results in the conversion of specific glutamic acid residues to gamma-carboxyglutamic acid in precursor proteins *i.e.*, VKDPs. In addition, there is increasing evidence that K_1_ and MK-4 have additional actions outside their role as enzymatic cofactor, e.g., inflammation, oxidative stress. In recent years, VK has been associated with a number of age-related conditions such as osteoporosis [1], the calcification process [3], insulin resistance [4,38], cancer [39], and cognitive decline [11]. This report aimed to investigate the influence of lifetime dietary VK exposure on the tissue content and distribution of the K_1_ and MK-4 vitamers at different life stages in male and female rats fed different diets since weaning, or subjected to caloric restriction, a life-prolonging model.

### 4.1. Study 1

In the first study, conducted in 3-, 12- and 22-month-old male and female rats fed different K_1_ diets since weaning, body weights were found to increase with age in both sexes but were not significantly affected by lifetime K_1_ intakes. Body weights have been shown to increase during aging in rodents and our results concur with previous studies [40,41]. The lack of effect of K_1_ intakes on body weights is of interest given the recently proposed role of VK in energy metabolism. In a series of studies using murine models, a specific form of the VKDP osteocalcin has been shown to modulate glucose and insulin metabolism, energy expenditure, and fat mass [4]. Although the endocrine role of osteocalcin remains an intense subject of debate, other studies conducted in rats [42] and humans [38,43] point to potential beneficial effects of increased VK intakes on metabolic profile and body weights. The trend towards lower body weights observed at 22 months in both males and females fed the H diet goes along those lines.

Plasma K_1_ increased as a function of diet in both sexes, however, no MK-4 could be detected under our experimental conditions. The presence of MK-4 in the circulation in response to K_1_ intake has been variable, some studies reporting low [6,27,28] or undetectable [8] values. Tissue contents and distribution of the K vitamers were found to differ, with K_1_ as the predominant vitamer in some organs *i.e.*, the liver, heart, and MK-4 in others *i.e.*, testes and brain. Such differential distributions of the K vitamers were observed in previous short-term studies [6,8,26,29]. One notable finding of the present study, however, is the large amount of total VK observed in ovaries, this organ being rich in both K_1_ and MK-4 vitamers. High levels of VK have been reported in a previous study [29], although levels were significantly lower than those reported here. Differences in strain (Brown–Norway *vs.* Sprague–Dawley) and VK content of the diets could explain the differences between the two studies. However, the high VK content of ovaries is intriguing as this organ is not known to be particularly rich in either of the known VKDPs. Whether the K vitamers possess actions in the ovary beyond their role in the carboxylation reaction remains to be determined; however, in a series of older studies, VK was shown to possess estrogenic activity. Specifically, treatment of castrate mice with either K_1_ and menadione resulted in increases in uterine weights and cornification of the vaginal epithelium [44].

Increasing K_1_ intake generally resulted in higher tissue K_1_ and MK-4, differences being mainly observed between the L and H, and the A and H groups suggesting that intakes >500 µg K_1_/kg diet are needed for tissue accumulation. These results are in line with those of previous studies conducted in normal [26] and germ-free [45] rats. In the great majority of organs, total VK tissue contents were higher in females than in males, the effect being mainly seen at 3 and 12 months of age. This finding has been observed by others [29,30] and is likely explained by the facilitatory action of estrogens on the intestinal absorption of K_1_ [46]. As discussed below, this estrogenic effect on VK absorption could contribute to the greater resistance of female rats to VK deficiency. Concentrations of the K vitamers and total VK were also affected by age in a number of organs (*i.e.*, the liver, spleen, brain, ovaries and testes), differences varying according to organs, sex (when applicable), and dietary groups. Except for ovaries for which VK contents increased throughout life, total VK concentrations either increased between 3 and 12 months or were comparable, and decreased in the second year of life. In 3-, 12- and 24-months old Brown–Norway rats which had been fed a nonpurified diet (NIH 31 M), liver K_1_ was found to be increased and MK-4 to be decreased in the heart, kidneys, lung, and cerebellum of old animals [29]. In a more recent study conducted in Fisher 344 male rats aged two, 12, and 24 months, a 28-day administration of a low K_1_ containing diet (~200 µg K_1_/kg diet) was associated with higher MK-4 concentrations in the liver, spleen, kidneys, heart, and cortex (myelin) in 24- compared to two-month-old rats; the opposite effect was observed for testes [28]. Divergent results among studies could be explained by differences in experimental design *i.e.*, VK contents of the diets, duration of exposure, *etc.*, and strain of animals.

In light of the growing importance of MK-4 in various physiological functions and given that the aging process can affect enzymatic activities [32,47] which could include those involved in the K_1_ to MK-4 conversion, we assessed the relative MK-4 to total VK tissue content (MK-4/total VK) in all organs. In contrast to what was observed for K_1_, MK-4, and total VK, the MK-4/total VK ratios were much less affected by sex, diet, and age. Regarding the latter, an interesting trend was observed. In high MK-4 containing organs such as the brain, testes, kidneys and ovaries, the high MK-4/total VK ratios were maintained in old age, this finding being observed for both sexes and in most dietary groups. In organs with lower MK-4 contents *i.e.*, liver, heart and spleen, MK-4/total VK ratios tended to increase with age in both sexes and dietary groups. Hence, irrespective of an organ’s usual MK-4 content, the proportion of this vitamer relative to total VK appears to increase with age. Whether this trend reflects an increased physiological demand for MK-4 in handling age-related conditions such as inflammation and oxidative stress remains to be established, but such hypothesis should be pursued in future studies.

Male rats have long been shown to be less resistant to VK deficiency compared to females [30,48,49]. The increased PT and APTT observed in young male rats fed the L diet corroborate these observations and confirm earlier findings that diets containing <500 µg K_1_/kg diet are insufficient to maintain normal coagulation [34]. It should be mentioned that, despite their increased coagulation times, no rat presented signs of bleeding or died due to hemorrhage.

### 4.2. Study 2

By virtue of its nature, the CR dietary regimen resulted in significantly lower body weights in old CR compared to *AL* groups. As a corollary to this and because the diet fed the CR rats was adjusted to contain equimolar amounts of vitamins and minerals as that fed the *AL* group, K_1_ intakes of CR rats were significantly higher than those fed the *AL* diet when expressed per unit body weight (Table 3). As shown in Table 4, the dietary regimen had no impact on the K vitamer profile in plasma and tissues, organs rich in K_1_ (*i.e.*, liver, heart) and MK-4 (*i.e.*, brain, testes) being those observed in study 1. Likewise, ovaries were found to contain high quantities of both K vitamers and the highest VK content of all organs. The general trend for higher VK tissue concentrations in females than in males was also observed, the effect being particularly marked for the MK-4 vitamer. Age had little effect on K_1_ tissue contents in contrast to MK-4, which were generally increased at 20 months compared to 3 months. Interestingly, despite the fact that rats fed the CR regimen consumed more K_1_ per unit body weight than their *AL* counterparts since adulthood, their tissue K_1_ and MK-4 contents at 20 months tended to be decreased when compared to those of the *AL* group. Lower tissue MK-4 content could be the result of lower synthesis from K_1_ or greater tissue utilization. Although assessing the activity of the UBIAD1 enzyme would have provided useful information regarding tissues’ synthetic capacities, this hypothesis is unlikely given that the MK-4/total VK ratios at 20 months were largely unaffected by CR. The second hypothesis probably represents a more plausible explanation as the aging process is characterized by molecular and cellular events that leads to impaired function and increased vulnerability to death [47,50]. In fact, a study conducted in 4-, 19- and 28-month-old rats aiming at assessing the impact of CR on α-tocopherol tissue distribution point in this direction. Compared to tissues of 19-month-old *AL* rats, α-tocopherol concentrations in CR rats were found to be lower by 48% in plasma, 30% in kidneys, 60% in the liver, and 44% in the heart homogenate. These results were interpreted as reflecting a greater utilization of tissue α-tocopherol in limiting the age-induced oxidative damage to membranes [51].

Finally, in this study, coagulation times were increased in old male rats fed the *AL* diet (PT, *p* < 0.05; trend for APTT). The fact that animals subjected to CR were able to maintain a more youthful coagulation profile *i.e.*, similar to that of 3-month-old animals, supports the notion that this dietary regimen is associated with a greater utilization of the K vitamers.

## 5. Conclusions

Dietary K_1_ intakes around the minimal amount required for normal blood coagulation had no significant influence on body weights of both male and female rats at different life stages. Tissue contents of the K vitamers differed according to organs, were generally higher in females than in males, and increased with K_1_ intake. The MK-4/total VK ratios tended to increase with age, possibly reflecting an increased physiological demand for MK-4 during aging. Our study also confirmed the greater susceptibility of male rats to VK insufficiency, notably at a younger age.

Despite lifelong higher K_1_ intakes per unit body weight, tissue K_1_ and MK-4 contents at 20 months were generally lower in CR rats compared to their *AL* counterparts. Whether the lower tissue MK-4 content is the result of lower synthesis from K_1_ or greater tissue utilization remains to be determined. However, the more youthful coagulation profile observed in old CR rats (*vs. AL* rats) tends to support the notion that CR is associated with greater utilization of the K vitamers to sustain physiological functions.

## Figures and Tables

**Table 1:** Daily food (g/100 g body weight (BW)) and K_1_ (µg/100 g·BW) intakes of 3-, 12-, and 22-month-old male (M) and female (F) Sprague–Dawley rats fed the low (L), adequate (A) or high (H) diets throughout their lives.

Age (Months)	Diet	Food Intake (g/100 BW/Day)		K_1_ Intake (µg/100 g·BW/Day)	
		F	M	F	M
3	L	5.7 ± 0.2	4.5 ± 0.1	562.6 ± 15.9	444.6 ± 9.4
	A	5.6 ± 0.2	4.5 ± 0.1	2780.4 ± 83.0 ^c^	2204.8 ± 56.2 ^c^
	H	6.3 ± 0.1 ^a,b^	4.7 ± 0.2	9433.2 ± 164.3 ^a,b^	7022.3 ± 217.7 ^a,b^
12	L	4.2 ± 0.2	3.0 ± 0.1	414.9 ± 15.3	297.2 ± 11.6
	A	4.2 ± 0.2	3.2 ± 0.1	2063.6 ± 101.2 ^c^	1574.8 ± 51.4 ^c^
	H	3.8 ± 0.2	3.0 ± 0.1	5987.3 ± 174.2 ^a,b^	4386.0 ± 165.9 ^a,b^
22	L	2.6 ± 0.2	2.7 ± 0.1	253.6 ± 14.4	268.9 ± 9.9
	A	2.5 ± 0.2	2.6 ± 0.1	1231.5 ± 85.6^c^	1281.9 ± 43.5 ^c^
	H	2.8 ± 0.2	2.7 ± 0.2	4141.4 ± 245.1 ^a,b^	3954.1 ± 229.3 ^a,b^

Based on the 3-way ANOVA analysis, food intakes decreased with age in both sexes (*p* < 0.001) and were higher in F than in M at 3 and 12 but not at 22 months of age (*p* < 0.001). Food intakes did not vary according to diet in M in contrast to F where, at 3 months, food intakes were significantly higher in the H than L and A groups (*p* < 0.05). Phylloquinone intakes decreased with age irrespective of diet in both sexes (*p* < 0.001) and were higher in F than in M at 3 and 12 but not at 22 months of age (*p* < 0.001). Phylloquinone intakes were significantly different between each dietary group, in both sexes (*p* < 0.001). The effect of diet was further tested within each sex and age group by one-way ANOVA’s followed by Tukey’s *post hoc* tests; ^a^: *p* < 0.05 between H and L group same age and sex; ^b^: *p* < 0.05 between H and A group same age and sex; ^c^: *p* < 0.05 between A and L group same age and sex. Values are expressed as mean ± SEM; *n* for each group are as in Figure 1.

**Table 2:** Plasma and tissue (liver, heart, kidneys, spleen, brain, ovaries and testes) K_1_, MK-4 and total (K_1_ + MK-4) vitamin K (VK) concentrations of 3-, 12-, and 22-month-old M and F Sprague–Dawley rats fed the L, A or H diets throughout their lives.

Organ (Months)	VK Diet (µg)	K1		MK-4	K1 + MK4		
		F	M	F	M	F	M
		pmol/g or L Plasma					
Plasma							
3	L	0.14 ± 0.11	0.04 ± 0.09	nd	nd	0.14 ± 0.11	0.04 ± 0.09
	A	0.21 ± 0.08	0.27 ± 0.17	nd	nd	0.21 ± 0.08	0.27 ± 0.17
	H	0.67 ± 0.35 ^a,b^	0.41 ± 0.30 ^a^	nd	nd	0.67 ± 0.35 ^a,b^	0.41 ± 0.30 ^a^
12	L	0.36 ± 0.29	0.21 ± 0.08	nd	nd	0.36 ± 0.29	0.21 ± 0.08
	A	0.44 ± 0.08	0.15 ± 0.08	nd	nd	0.44 ± 0.08	0.15 ± 0.08
	H	1.83 ± 0.53 ^a,b^	0.76 ± 0.43 ^a,b^	nd	nd	1.83 ± 0.53 ^a,b^	0.76 ± 0.43 ^a,b^
22	L	0.96 ± 0.35	0.84 ± 0.97	nd	nd	0.96 ± 0.35	0.84 ± 0.97
	A	1.83 ± 0.58	1.19 ± 0.42	nd	nd	1.83 ± 0.58	1.19 ± 0.42
	H	4.60 ± 1.63 ^a,b^	2.29 ± 1.08 ^a^	nd	nd	4.60 ± 1.63 ^a,b^	2.29 ± 1.08 ^a^
Liver							
3	L	46.4 ± 4.9	43.0 ± 13.9	26.7 ± 2.6	16.4 ± 3.5	73.1 ± 6.3	59.5 ± 16.4
	A	49.8 ± 7.7	68.6 ± 25.7	37.4 ± 7.2	10.2 ± 3.2	87.2 ± 12.5	78.8 ± 27.3
	H	199.6 ± 30.0 ^a,b^	73.3 ± 19.5	135.5 ± 14.6 ^a,b^	14.5 ± 2.9	335.1 ± 32.7 ^a,b^	87.8 ± 17.7
12	L	71.5 ± 14.3	33.7 ± 7.8	35.8 ± 7.4	23.6 ± 7.7	107.3 ± 20.7	57.4 ± 11.3
	A	171.9 ± 43.8 ^c^	44.3 ± 6.8	68.4 ± 10.9	31.9 ± 7.6	240.3 ± 41.5 ^c^	76.2 ± 12.7
	H	410.3 ± 13.9 ^a,b^	122.5 ± 37.6 ^a^	142.7 ± 10.9 ^a^	38.9 ± 11.8	553.0 ± 22.4 ^a^	161.5 ± 43.4 ^a^
22	L	34.7 ± 2.9	25.6 ± 5.4	65.0 ± 9.3	57.5 ± 8.8	99.7 ± 8.1	83.1 ± 13.7
	A	69.2 ± 12.6 ^c^	52.6 ± 8.5	81.0 ± 17.9	58.8 ± 7.8	150.2 ± 21.7	111.4 ± 8.3
	H	191.1 ± 14.3 ^a,b^	79.1 ± 19.1 ^a^	104.4 ± 18.0	61.7 ± 16.0	295.4 ± 29.9 ^a^	140.8 ± 25.8
Heart							
3	L	13.2 ± 1.7	9.5 ± 0.3	19.5 ± 2.2	9.7 ± 1.6	32.7 ± 3.2	19.2 ± 1.7
	A	32.1 ± 2.1 ^c^	31.2 ± 1.9 ^c^	25.0 ± 2.4	26.6 ± 5.2 ^c^	57.1 ± 2.9 ^c^	57.7 ± 5.6 ^c^
	H	300.4 ± 74.4 ^a,b^	188.1 ± 42.1 ^a,b^	265.9 ± 60.2 ^a,b^	59.9 ± 11.5 ^a,b^	566.2 ± 134.4 ^a,b^	248.0 ± 52.6 ^a,b^
12	L	16.3 ± 0.7	15.6 ± 5.3	17.7 ± 2.7	34.7 ± 20.9	34.0 ± 3.4	50.2 ± 26.0
	A	41.5 ± 6.4 ^c^	32.1 ± 4.0 ^c^	27.7 ± 4.3	15.7 ± 3.6	69.2 ± 10.4 ^c^	47.7 ± 7.4
	H	225.6 ± 32.0 ^a,b^	213.9 ± 42.6 ^a,b^	165.9 ± 24.6 ^a,b^	79.6 ± 11.4 ^a,b^	391.5 ± 51.9 ^a,b^	293.4 ± 52.9 ^a,b^
22	L	30.8 ± 4.7	30.1 ± 2.8	56.9 ± 3.5	43.5 ± 3.8	87.6 ± 5.1	73.6 ± 3.1
	A	54.9 ± 7.3 ^c^	47.1 ± 7.7	62.5 ± 7.7	42.0 ± 8.1	117.4 ± 12.1	89.1 ± 15.4
	H	106.3 ± 14.4 ^a,b^	100.9 ± 39.0 ^a^	104.0 ± 24.1	89.7 ± 25.8	210.2 ± 37.1 ^a,b^	190.6 ± 56.1
Kidneys							
3	L	2.8 ± 0.5	4.7 ± 0.6	17.6 ± 3.2	11.9 ± 3.5	20.4 ± 3.6	16.6 ± 3.2
	A	9.3 ± 4.3	6.5 ± 1.4	29.9 ± 2.9	14.7 ± 3.7	39.2 ± 4.9	21.1 ± 3.1
	H	18.7 ± 4.7	10.2 ± 2.2	176.7 ± 24.6 ^a^	34.9 ± 9.2	195.4 ± 28.1 ^a,b^	45.1 ± 10.9 ^a^
12	L	4.3 ± 1.1	3.7 ± 0.8	28.5 ± 8.8	12.1 ± 3.8	32.8 ± 9.5	15.8 ± 4.4
	A	6.0 ± 1.2	6.6 ± 1.1	32.6 ± 8.4	22.1 ± 5.8	38.6 ± 9.6	28.8 ± 6.8
	H	32.0 ± 4.6 ^a,b^	6.4 ± 1.8	154.1 ± 34.8 ^a,b^	32.2 ± 8.5 ^a^	186.1 ± 30.3 ^a,b^	38.6 ± 8.9 ^a^
22	L	7.7 ± 0.9	3.6 ± 0.5	30.8 ± 4.5	26.3 ± 3.0	38.5 ± 4.7	30.0 ± 3.1
	A	11.6 ± 1.1	7.8 ± 0.6 ^c^	52.4 ± 7.1	38.9 ± 3.7	63.9 ± 7.6 ^c^	46.7 ± 4.2
	H	30.6 ± 9.8 ^a,b^	20.3 ± 5.5 ^a,b^	42.3 ± 8.6	19.4 ± 8.4	72.9 ± 13.3 ^a^	39.7 ± 12.8
Spleen							
3	L	16.7 ± 1.5	16.6 ± 2.3	17.2 ± 3.2	8.6 ± 1.9	33.9 ± 4.0	25.1 ± 3.3
	A	24.9 ± 4.4	28.0 ± 3.4 ^c^	23.6 ± 6.8	17.1 ± 3.9	48.5 ± 9.6	45.1 ± 4.7
	H	80.1 ± 17.8 ^a,b^	49.4 ± 6.0 ^a,b^	73.1 ± 15.3 ^a,b^	37.6 ± 4.2 ^a,b^	153.2 ± 28.7 ^a,b^	86.9 ± 5.1 ^a^
12	L	15.5 ± 1.8	15.5 ± 6.8	17.5 ± 3.5	11.4 ± 4.4	33.0 ± 5.2	26.8 ± 10.6
	A	30.4 ± 4.5 ^c^	31.8 ± 10.4	50.9 ± 11.9	47.2 ± 17.1	81.3 ± 15.2	79.0 ± 26.6
	H	110.9 ± 17.7 ^a,b^	38.2 ± 7.0	66.8 ± 8.7	35.7 ± 12.5	177.6 ± 22.8 ^a^	73.8 ± 18.2 ^a^
22	L	18.8 ± 3.9	22.7 ± 6.0	43.1 ± 10.6	56.3 ± 14.4	61.8 ± 10.8	79.0 ± 19.4
	A	28.4 ± 4.7	26.1 ± 3.8	67.1 ± 8.9	48.3 ± 7.8	95.5 ± 13.2	74.3 ± 11.1
	H	91.9 ± 14.9 ^a,b^	126.7 ± 17.2 ^a,b^	101.5 ± 21.1 ^a^	120.6 ± 22.1	193.4 ± 31.1 ^a,b^	247.2 ± 35.9 ^a,b^
Brain							
3	L	2.0 ± 0.7	2.3 ± 0.4	54.2 ± 7.1	21.1 ± 1.6	56.2 ± 7.3	23.4 ± 1.3
	A	4.3 ± 0.8	3.7 ± 0.6	64.5 ± 4.8	47.2 ± 5.6 ^c^	68.8 ± 5.1	51.0 ± 5.7 ^c^
	H	8.0 ± 0.7 ^a,b^	8.4 ± 0.9 ^a,b^	197.5 ± 7.6 ^a,b^	144.3 ± 12.0 ^a,b^	205.4 ± 8.0 ^a,b^	152.6 ± 12.7 ^a,b^
12	L	1.6 ± 0.3	2.6 ± 1.2	47.1 ± 4.1	46.9 ± 18.9	48.7 ± 4.0	49.5 ± 20.1
	A	4.2 ± 0.4 ^c^	3.3 ± 0.5	71.9 ± 7.1 ^c^	40.6 ± 3.9	76.1 ± 7.3 ^c^	43.9 ± 4.4
	H	7.4 ± 1.2 ^a,b^	9.1 ± 2.3 ^a^	187.2 ± 20.8 ^a,b^	157.6 ± 10.5 ^a,b^	194.6 ± 20.7 ^a,b^	166.7 ± 12.4 ^a,b^
22	L	2.2 ± 0.3	3.9 ± 0.2	53.5 ± 5.9	56.8 ± 11.9	55.7 ± 6.0	60.6 ± 11.8
	A	3.2 ± 0.2	3.4 ± 0.4	66.7 ± 7.9	85.5 ± 4.1	70.0 ± 8.0	88.9 ± 4.2
	H	4.4 ± 0.8 ^a^	5.4 ± 1.7	70.0 ± 8.5	63.3 ± 20.5	74.4 ± 9.0	68.7 ± 21.9
Ovaries							
3	L	89.1 ± 35.9		190.6 ± 39.1		279.7 ± 34.4	
	A	133.3 ± 19.2		296.1 ± 100.0		429.5 ± 98.5	
	H	267.6 ± 42.4 ^a^		616.4 ± 48.2 ^a^		884.0 ± 89.7 ^a,b^	
12	L	60.0 ± 22.1		187.1 ± 39.9		247.1 ± 54.7	
	A	218.2 ± 9.2 ^c^		517.2 ± 93.4 ^c^		735.4 ± 99.8 ^c^	
	H	424.4 ± 46.6 ^a^		762.1 ± 67.3 ^a^		1186.5 ± 111.2 ^a^	
22	L	183.9 ± 32.3		516.2 ± 72.2		700.1 ± 99.4	
	A	307.8 ± 50.2		706.6 ± 90.7		1014.4 ± 138.4	
	H	416.6 ± 46.7		656.7 ± 49.6		1073.3 ± 51.8	
Testes							
3	L		13.4 ± 4.5		63.8 ± 7.2		77.3 ± 6.6
	A		7.2 ± 1.4		134.5 ± 15.8 ^c^		141.6 ± 16.3 ^c^
	H		11.8 ± 3.6		292.4 ± 30.0 ^a,b^		304.1 ± 28.6 ^a,b^
12	L		9.9 ± 4.8		83.4 ± 28.3		93.3 ± 29.4
	A		20.9 ± 12.8		258.7 ± 43.6 ^c^		279.7 ± 51.4 ^c^
	H		22.0 ± 11.8		299.1 ± 52.3 ^a^		321.1 ± 45.0 ^a^
22	L		4.5 ± 0.6		64.3 ± 8.1		68.8 ± 8.2
	A		6.8 ± 0.6		96.3 ± 8.3 ^c^		103.1 ± 8.7 ^c^
	H		13.3 ± 1.9 ^a,b^		81.4 ± 9.5		94.7 ± 9.7

Main sex, diet and age effects were assessed by conducting 3-way ANOVAs followed by pairwise multiple comparisons; results for these analyses are presented in the text (Section 3.1.2). The effect of diet on plasma and tissue K_1_, MK-4 and total VK concentrations were further tested within each sex and age group by one-way ANOVA’s followed by Tukey’s *post hoc* tests; ^a^: *p* < 0.05 between H and L groups same age and sex, ^b^: *p* < 0.05 between H and A groups same age and sex, ^c^: *p* < 0.05 between A and L groups same age and sex. Values are expressed as mean ± SEM; *n* = 5–7/sex/age/diet groups. MK-4: menaquinone-4. nd: non-detectable.

**Table 3:** Body weights, daily food (g/day and g/100 g·BW/day), and K_1_(µg/day and µg/100 g·BW) intakes of 3- and 20-month-old M and F Sprague–Dawley rats fed an *ad libitum* (*AL*) or calorie restricted (CR) diet.

Age (Months)	Diet	Body Weight (g)		Food Intake (g/Day)		K_1_ Intake (µg/Day)		Food Intake (g/100 g·BW/Day)		K_1_ Intake (µg/100 g·BW/Day)	
		F	M	F	M	F	M	F	M	F	M
3	*AL*	277 ± 35 ^z^	523 ± 73	16.5 ± 1.7 ^z^	22.5 ± 1.3	8.3 ± 0.8 ^z^	11.2 ± 0.6	6.0 ± 0.5 ^z^	4.3 ± 0.4	3.0 ± 0.2 ^z^	2.2 ± 0.2
20	*AL*	647 ± 171 ^y,z^	939 ± 132 ^y^	18.0 ± 3.5 ^z^	27.1 ± 3.8 ^y^	9.0 ± 1.8	13.6 ± 9.3	2.9 ± 0.5 ^y^	2.9 ± 0.4 ^y^	1.4 ± 0.2 ^y^	1.4 ± 0.2^y^
	CR	281 ± 29 ^z,^*	484 ± 37 *	11.0 ^x,z^^,^*	16.0 ^x,^*	9.2 ^z^	13.3	4.6 ^x,z,^*	3.9 *	3.8 ^x,z,^*	3.3 ^x,^*

In all age and diet groups, body weights were higher in M than F (*p* < 0.05). In both sexes fed the *AL* diet, body weights were significantly increased at 20 months (*vs.* 3 months) and significantly decreased in 20 CR rats (*vs.* 20 *AL*) (*p* < 0.05). Food intakes expressed as g/day were significantly higher in M than F in all age and diet groups, and were higher in 20 *AL vs.* 3 groups (*p* < 0.05). As expected, food intakes were lower in the 20 CR than in 20 *AL* groups (both sexes; *p* < 0.05). Phylloquinone intakes µg/day were significantly higher in M than F (3 and 20 CR) but were similar among age and diet groups (*p* < 0.05). When expressed per 100 g·BW, food and K_1_ intakes (µg/100 g·BW/day) were higher in F than M (3 and 20 CR groups), were lower in older animals fed the *AL* diet (*vs.* 3 months), and were higher in the 20 CR *vs.* 20 *AL* groups (*p* < 0.05); ^x^: *p* < 0.05 between 20 CR and 3-month group, same sex; ^y^: *p* < 0.05 between 20 *AL* and 3-month group, same sex; ^z^: *p* < 0.05 between M and F, same age and diet; *: *p* < 0.05 between 20 *AL* and 20 CR, same sex. Values are expressed as mean ± SEM; *n* = 6–8/sex/age/diet groups.

**Table 4:** Plasma and tissue (the liver, heart, kidneys, spleen, brain, ovaries and testes) K_1_, MK-4 and total VK (K_1_ + MK-4) concentrations of 3- and 20-month-old M and F Sprague–Dawley rats fed an *AL* or CR diet.

Diet		K1		MK-4		K1 + MK4	
		F	M	F	M	F	M
		pmol/g or L Plasma		pmol/g or L Plasma		pmol/g or L Plasma	
Plasma							
3	*AL*	0.21 ± 0.03	0.25 ± 0.08	nd	nd	0.21 ± 0.03	0.25 ± 0.08
20	*AL*	1.73 ± 0.15 ^y,z^	1.12 ± 0.11 ^y^	nd	nd	1.73 ± 0.15 ^y,z^	1.12 ± 0.11 ^y^
	CR	1.35 ± 0.10 ^x,z,^*	0.85 ± 0.07 ^x,^*	nd	nd	1.35 ± 0.10 ^x,^*	0.85 ± 0.07 ^x,^*
Liver							
3	*AL*	54.2 ± 8.1	48.4 ± 17.9	43.4 ± 3.6 ^z^	18.5 ± 2.1	97.6 ± 7.7	56.9 ± 20.0
20	*AL*	71.2 ± 7.9	50.7 ± 6.1	66.6 ± 12.0	54.5 ± 6.1 ^y^	137.8 ± 17.9	105.2 ± 7.2 ^y^
	CR	49.0 ± 5.7 *	50.6 ± 6.6	61.6 ± 8.2	48.6 ± 5.6 ^x^	110.6 ± 10.5	99.2 ± 7.3 ^x^
Heart							
3	*AL*	32.1 ± 2.1	31.2 ± 1.9	24.7 ± 2.4	26.2 ± 5.1	56.7 ± 2.9	57.4 ± 5.5
20	*AL*	47.2 ± 6.4	46.9 ± 5.1	55.5 ± 5.6 ^y^	43.3 ± 5.6	102.8 ± 10.2 ^y^	90.2 ± 9.6 ^y^
	CR	32.7 ± 6.8	41.5 ± 5.8	53.6 ± 4.8 ^x,z^	38.7 ± 4.6	86.3 ± 9.0	80.2 ± 7.4
Kidney							
3	*AL*	9.3 ± 4.3	5.6 ± 1.4	29.5 ± 2.8 ^z^	12.0 ± 3.3	38.7 ± 4.9 ^z^	17.6 ± 3.5
20	*AL*	11.8 ± 1.1 ^z^	7.6 ± 0.4	54.5 ± 4.7 ^y^^,z^	35.0 ± 3.9 ^y^	66.3 ± 5.1 ^y,z^	42.6 ± 4.3 ^y^
	CR	10.1 ± 1.0 ^z^	6.2 ± 0.4 *	45.3 ± 4.7 ^z^	33.2 ± 1.6 ^x^	55.4 ± 5.0 ^z^	39.4 ± 1.5 ^x^
Spleen							
3	*AL*	23.6 ± 5.1	25.1 ± 2.3	17.0 ± 3.0	19.7 ± 3.7	40.6 ± 7.0	44.8 ± 5.3
20	*AL*	26.9 ± 3.2	23.5 ± 2.2	64.0 ± 7.2 ^y,z^	41.1 ± 4.4 ^y^	90.9 ± 8.9 ^y,z^	64.6 ± 6.3
	CR	24.2 ± 3.0	23.0 ± 3.0	53.7 ± 6.0 ^x^	42.8 ± 5.7 ^x^	77.9 ± 7.8 ^x^	65.7 ± 7.2
Brain							
3	*AL*	4.3 ± 0.8	3.7 ± 0.6	63.6 ± 4.7 ^z^	46.6 ± 5.5	67.9 ± 5.0 ^z^	50.3 ± 5.6
20	*AL*	3.5 ± 0.4	3.6 ± 0.4	69.9 ± 5.6	66.3 ± 7.3	73.4 ± 5.8	70.0 ± 7.3
	CR	4.6 ± 0.4	3.9 ± 0.4	52.9 ± 5.8 *	54.8 ± 7.2	57.4 ± 5.9	58.7 ± 7.4
Ovaries							
3	*AL*	133.7 ± 19.6		426.5 ± 114.9		560.2 ± 133.1	
20	*AL*	278.5 ± 27.4 ^y^		636.3 ± 58.5		914.8 ± 76.7 ^y^	
	CR	240.5 ± 28.0 ^x^		453.4 ± 42.1 *		693.9 ± 65.0 *	
Testes							
3	*AL*		6.1 ± 1.0		131.8 ± 19.0		137.9 ± 19.6
20	*AL*		6.1 ± 0.6		110.4 ± 7.8		116.5 ± 7.8
	CR		4.6 ± 0.3		123.6 ± 8.9		128.2 ± 8.7

Age and diet effects were determined in M and F by conducting distinct one-way ANOVA’s followed by Tukey’s *post hoc* tests. Student’ *t*-test was used to further assess the effect of CR at 20 months, and that of sex *i.e.*, given age and diet group. ^x^: *p* < 0.05 between 20 CR and 3-months, same sex; ^y^: *p* < 0.05 between 20 *AL* and 3 months, same sex; ^z^: *p* < 0.05 between M and F, same age and diet; *: *p* < 0.05 between 20 *AL* and 20 CR, same sex. Values are expressed as mean ± SEM; *n* = 6–8/sex/age/diet groups.

**Table 5:** Prothrombin (PT) and activated partial thromboplastin (APTT) times of 3- and 20-month-old M and F Sprague–Dawley rats fed an *AL* or CR diet.

Age (Months)	Diet	PT		APTT	
		(s)			
		M	F	M	F
3	*AL*	14.5 ± 1.2	13.5 ± 0.3	20.8 ± 1.2	21.6 ± 1.3
20	*AL*	12.8 ± 0.2	12.8 ± 0.3	21.7 ± 1.4	20.0 ± 1.6
	CR	14.0 ± 0.4 ^z,^*	12.9 ± 0.2	23.3 ± 1.6	20.3 ± 1.1

At 20 months, the mean PT value of M fed the CR diet was statistically higher than that of the 20 *AL* group (*p* < 0.05), and comparable to that of male rats aged 3 months. A sex effect was observed in 20-month-old rats fed the CR diet, M showing a statistically higher mean value than F (*p* < 0.05). ^x^: *p* < 0.05 between 20 CR and 3-months, same sex; ^y^: *p* < 0.05 between 20 *AL* and 3 months, same sex; ^z^: *p* < 0.05 between M and F, same age and diet; *: *p* < 0.05 between 20 *AL* and 20 CR, same sex. Values are expressed as mean ± SEM; *n* = 6–8/sex/age/diet groups.
